# From river blindness control to elimination: bridge over troubled water

**DOI:** 10.1186/s40249-018-0406-7

**Published:** 2018-03-28

**Authors:** Robert Colebunders, Maria-Gloria Basáñez, Katja Siling, Rory J. Post, Anke Rotsaert, Bruno Mmbando, Patrick Suykerbuyk, Adrian Hopkins

**Affiliations:** 1 Global Health Institute, University of Antwerp, Antwerp, Belgium; 2 London Centre for Neglected Tropical Disease Research, Imperial College London, London, UK; 3 Institute of Tropical Medicine, Antwerp, Belgium; 4 London School of Hygiene & Tropical Medicine, London, UK; 5 Liverpool John Moores University, Liverpool, UK; 6 National Institute for Medical Research, Tanga, Tanzania; 7 Neglected and Disabling diseases of Poverty Consultant, Gravesend, Kent, UK

**Keywords:** Onchocerciasis, Control, Elimination, Monitoring & evaluation, Community drug distributors, Epilepsy, Prevalence, Incidence

## Abstract

**Background:**

An estimated 25 million people are currently infected with onchocerciasis (a parasitic infection caused by the filarial nematode *Onchocerca volvulus* and transmitted by *Simulium* vectors), and 99% of these are in sub-Saharan Africa. The African Programme for Onchocerciasis Control closed in December 2015 and the World Health Organization has established a new structure, the Expanded Special Project for the Elimination of Neglected Tropical Diseases for the coordination of technical support for activities focused on five neglected tropical diseases in Africa, including onchocerciasis elimination.

**Aims:**

In this paper we argue that despite the delineation of a reasonably well-defined elimination strategy, its implementation will present particular difficulties in practice. We aim to highlight these in an attempt to ensure that they are well understood and that effective plans can be laid to solve them by the countries concerned and their international partners.

**Conclusions:**

A specific concern is the burden of disease caused by onchocerciasis-associated epilepsy in hyperendemic zones situated in countries experiencing difficulties in strengthening their onchocerciasis control programmes. These difficulties should be identified and programmes supported during the transition from morbidity control to interruption of transmission and elimination.

**Electronic supplementary material:**

The online version of this article (10.1186/s40249-018-0406-7) contains supplementary material, which is available to authorized users.

## Multilingual abstracts

Please see Additional file 1 for translations of the abstract into the five official working languages of the United Nations.

## Background

According to the World Health Organization (WHO), at least 25 million people are currently infected with onchocerciasis (a parasitic infection caused by the filarial nematode *Onchocerca volvulus*), and 123 million people, 99% of them in sub-Saharan Africa, live in areas that put them at risk of infection [1, 2]. The parasite is transmitted by *Simulium* (blackfly) vectors which breed in fast-flowing waters, from which arises the name by which the disease is best known: River Blindness. As a consequence of onchocerciasis, before the inception of the African Programme for Onchocerciasis Control (APOC) in 1995, 10 million people suffered from its dermatological manifestations, with > 400 000 of them blind and 900 000 visually impaired [3]. Studies have also reported a significant association between onchocerciasis and excess human mortality [4, 5], as well as between onchocerciasis and epilepsy [6–8]. Despite this, the proportion of persons suffering from onchocerciasis-associated epilepsy remains to be determined.

Major efforts to control River Blindness started with the establishment of the Onchocerciasis Control Programme in West Africa (OCP) in 1974. Through at least 14 years of weekly aerial spraying with larvicidal insecticides of the simuliid vectors’ riverine breeding sites, this programme succeeded in eliminating transmission (and hence the parasite) in virtually all of the ‘core’ savannah areas of the seven initial OCP countries [9, 10]. Subsequently, by integrating this vector control with yearly mass distribution of the (broad-spectrum) anti-parasitic drug ivermectin, onchocerciasis was also eliminated as a disease of public health importance from 10 of the final 11 West African OCP countries by the time the programme closed in 2002 [11]. In some OCP foci, onchocerciasis was eliminated by (annual or biannual) mass drug administration of ivermectin even in the absence of vector control [12, 13].

Ivermectin (Mectizan®), a safe and efficacious anthelmintic with effects on the microfilarial stages (among others) of the parasite, was registered for onchocerciasis control in 1987, and is being donated by Merck Sharp & Dohme, MSD (known as Merck & Co. Inc. in the USA and Canada) for use in Africa, Latin America and Yemen for as long as necessary to eliminate the disease as a public health problem [14]. To take advantage of this donation, many eye-care non-governmental organizations (NGOs) working with the governments of endemic countries began mass ivermectin treatment in the most heavily infected communities and particularly in Africa [15, 16]. APOC was established in 1995 to co-ordinate and extend these activities using Community-Directed Treatment with Ivermectin (CDTI) as its main strategy to increase ivermectin coverage, and it aimed to control onchocerciasis in 20 endemic countries outside the OCP [2, 17]. Before the start of APOC in 1995, 32 million people were infected with onchocerciasis, with > 100 million of these at risk [3]. By 2014 (1 year before APOC’s closure), 112 million people were benefitting from CDTI, which averted annually the loss of 2 million Disability-Adjusted Life Years (DALYS) at a cost of only US$27 per DALY averted, making it very cost-effective [3]. In 2010, APOC shifted its focus from control of the disease to its elimination [18] and, in 2012, the WHO, in its roadmap for “Accelerating work to overcome the global impact of neglected tropical diseases” (NTDs), set the goals of eliminating onchocerciasis in selected African countries by 2020 [19]. Also in 2012, APOC’s Joint Action Forum expanded this goal to 80% of endemic countries with onchocerciasis eliminated by 2025 [20].

The aim of this paper is to argue that despite the development of a reasonably well-defined elimination strategy [21], its implementation will present difficulties in practice. We aim to highlight such difficulties, to try and ensure that they are well understood so that effective plans can be laid to solve them by the countries concerned and their international partners through technical support by the Expanded Special Project for the Elimination of Neglected Tropical Diseases (ESPEN), a newly created structure at WHO AFRO. A specific concern is the burden of onchocerciasis-associated disease that remains especially in hyperendemic zones situated in countries experiencing difficulties in strengthening their onchocerciasis control programmes. These difficulties need to be carefully identified and the programmes strongly supported during their transition from morbidity control to interruption of transmission and elimination.

### Expanded Special Project for the Elimination of Neglected Tropical Diseases (ESPEN)

APOC closed in December 2015 and WHO has established a new structure, the Expanded Special Project for the Elimination of Neglected Tropical Diseases (ESPEN) [22] for the coordination of technical support for five NTDs in Africa, including onchocerciasis elimination activities. The elimination efforts include extending ivermectin treatment to hypoendemic (previously excluded) and operationally challenging areas, and also implementing intensive surveillance. According to recent estimates, this could save US$1.5–1.6 billion over 2013–2045 compared to the scenario in which onchocerciasis is controlled but not eliminated [23]. The project’s plan includes the setting up of independent ‘national oversight committees’ for onchocerciasis elimination in all countries with onchocerciasis endemic foci, and recently in many countries such committees have been established. The committees operate under a variety of names (e.g. National Onchocerciasis Elimination Committee or NTD Technical Advisory Committee), and are in principle independent and advisory to the ministries of health, the decision makers which operate the national control programmes.

When the oversight committees are set up, their first task is to review the current epidemiological situation throughout the country. This often involves conducting new “elimination” prevalence surveys countrywide or in selected areas. Simultaneously, they try to define the so-called transmission zones in every part of the country (which will allow mapping of the hypoendemic areas where CDTI is to be instigated – usually twice a year) and identify sentinel sites for epidemiological and entomological surveillance. A transmission zone is a geographical area where transmission of *O. volvulus* occurs by locally breeding vectors and which can be regarded as a natural ecological and epidemiological unit for intervention [18]. As soon as possible, the committees start to assess progress towards elimination in all of the transmission zones, and this assessment includes prevalence surveys and examination of CDTI coverage. This process should identify programmatic insufficiencies in CDTI projects already operating in meso- and hyperendemic areas and result in appropriate corrective action. After the national elimination programmes have finished this initial review period, they are expected to settle into a new phase whereby the oversight committee considers annual progress reports from each transmission zone and recommends as necessary to the Ministry of Health action to reach or accelerate elimination.

The expert advisory committees should be able to consider a country’s progress towards elimination in much greater detail than was possible by APOC. For instance, the Uganda Onchocerciasis Elimination Expert Advisory Committee has already played a major role in guiding the interruption of transmission in 15 of the 17 Ugandan foci. However, Uganda is a small country whose committee is very inclusive, with district vector control officers attending committee meetings, presenting their reports and participating in discussions. Furthermore, in Uganda there has been a history of interest in onchocerciasis, and hence there already existed significant expertise within the Ministry of Health. Besides, Uganda has benefitted from the early and effective establishment of its advisory committee with the support of The Carter Center [24]. Many other countries (such as Liberia) have not had this historical head-start and may find it more difficult to make progress without significant external expertise and support. Also in large countries (Nigeria is an example) it will not be feasible to include Ministry of Health field operatives from all the districts as observers on the committee. ESPEN will have a role in the support of these expert committees which is mostly technical but in some cases also financial. However, ESPEN remains a small organisation and will not have the capacity to intervene directly in every endemic country.

Although the replacement of APOC by ESPEN has the potential advantage of generating a pan-African platform for integrated NTD control, the delays in organising ESPEN have also created some confusion and a temporary lack of direction. ESPEN has been mandated to cover five NTDs (onchocerciasis, lymphatic filariasis, schistosomiasis, soil-transmitted helminthiases and trachoma) with a budget that is far from generous. While it establishes itself, ESPEN has prioritised 14 countries for attention (Benin, Chad, Central African Republic, Comoros, Republic of Congo, Democratic Republic of the Congo, Ethiopia, Guinea, Guinea Bissau, Nigeria, São Tomé & Príncipe, South Sudan, Tanzania and Togo). However, these countries have been chosen from a consideration of all five NTDs, so several of the countries would not have been prioritised on the basis of onchocerciasis alone, and others which have significant onchocerciasis problems have not been included (such as Cameroon and Sierra Leone). This may restrict the budget available for effective onchocerciasis elimination efforts across the whole of Africa. A number of countries (such as the UK and the USA) have stepped in to try and fill the gaps by channelling direct country support through various organisations including NGOs (e.g., Sightsavers in the UK), but it is unclear whether this commitment will be sufficient and sustained until elimination. Whilst ESPEN’s onchocerciasis elimination plans include the expansion of CDTI to hypoendemic zones, there is a risk that the countries with already weak onchocerciasis control programmes may not receive the financial and technical support needed to implement and monitor onchocerciasis elimination programmes effectively.

### Eliminating onchocerciasis – The progress so far

The shift from control to elimination requires a major change in thinking, planning, funding and national support. In the absence of complementary vector control strategies [21], achieving good geographic and therapeutic ivermectin coverage as well as minimising systematic non-compliance are essential for onchocerciasis elimination. The former refers to the proportion of communities and individuals within communities treated, and the latter to the proportion of individuals that never take treatment. To control onchocerciasis as a public health problem, APOC recommended a minimum ivermectin therapeutic coverage of 65%; however, for elimination ≥80% therapeutic coverage and 100% geographical coverage will be needed as already recommended [25].

Epidemiological models suggest that to achieve elimination solely by means of mass ivermectin treatment, the minimum required therapeutic coverage of 65–80% of the total population (aged ≥5 years) (equivalent to 80–95% among those eligible) must be attained and sustained over a long period whose duration depends, partly, on the baseline level of onchocerciasis endemicity, measured by initial microfilarial prevalence and load [26–28]. The ONCHOSIM and EPIONCHO models predict that the provisional operational thresholds for treatment interruption and initiation of surveillance (pOTTIS), suggested by APOC (2010) [18], can be reached by annual CDTI (total coverage 80%) within 14–17 years for mesoendemic regions, but may require > 17 years (ONCHOSIM) or > 25 years (EPIONCHO) for highly hyperendemic (holoendemic) foci [28]. In both sets of simulations a 5% of systematic non-compliers was assumed and initial prevalences ranging from 50 to 90% were explored to cover the range from meso- to holoendemic onchocerciasis [29]. However, such predictions may not apply in all endemic areas in Africa because of a) different *Onchocerca*–*Simulium* complexes, particularly for forest onchocerciasis [30]; b) a possible greater proportion of non-adherence to treatment, particularly in loiasis co-endemic areas [31, 32], and c) differences between the magnitude of the pOTTIS and the true transmission breakpoints [28, 33]. The higher the initial endemicity, the lower the true elimination thresholds and, therefore, the less useful the current pOTTIS are as indicative of ultimate elimination. Currently the pOTTIS have been taken as a microfilarial prevalence < 1.4% 1 year after the last treatment round (a weighted mean of the values proposed by APOC 2010 [18]), but they would have to be much more stringent in areas of high baseline endemicity. In addition, the recent WHO [34] elimination guidelines do not advocate the measurement of microfilarial prevalence (by skin snips) among the metrics on which to make decisions about stopping treatment and verifying elimination.

APOC was set up in 1995 as a control (morbidity-reduction) programme, and following the Conference on Eradicability of Onchocerciasis in 2002 [35], it was considered doubtful that elimination could be achieved in Africa with mass administration of ivermectin alone. The arguments put forward included the large size of onchocerciasis endemic areas, the fact that these areas are often contiguous, and that the members of the *Simulium damnosum s.l.* complex are highly efficient in transmitting *O. volvulus*. The same conclusions were reached by other authors after assessing the empirical evidence available at the time regarding the impact of repeated ivermectin mass treatments on parasitological and transmission indices in West Africa [36]. At the time of that publication, data on 6-monthly treatments in Africa were sparse and did not allow conclusions to be drawn on the effectiveness of increased treatment frequency. However, elimination was thought to be possible in the Americas where onchocerciasis foci were often smaller and more circumscribed, and where some of the simuliid species involved in transmission have lower vector competence [37]. Indeed, the Onchocerciasis Elimination Program for the Americas (OEPA) has succeeded in eliminating onchocerciasis from Colombia, Ecuador, Guatemala, Mexico, and parts of Venezuela [38], using mostly biannual (6-monthly, semi-annual) treatment with ivermectin, and quarterly treatments in some foci [39].

Despite the challenges in achieving good treatment coverage in Africa, there is now a general belief that onchocerciasis elimination should be ultimately feasible in most, if not all, endemic areas and there is empirical evidence to support this notion. In 2005, a longitudinal study in three initially meso- to hyperendemic onchocerciasis foci (with strongly seasonal transmission by *S. sirbanum*) in Mali and Senegal, where ivermectin had been distributed for 15–17 years, documented no evidence of transmission over a 3–5-year period after stopping treatment [13]. Nevertheless, it should be noted that a recent evaluation study which covered some of the same areas (in the River Gambia focus of Senegal) found 7/279 children positive with antibodies for the Ov16 antigen (the marker of exposure/infection recommended by the recent WHO guidelines [34]). However, some of these results could be false positives and it is not yet clear whether this represents continuing autochthonous transmission or exposure to infective larvae through the bite of infective immigrant flies [40]. Of interest is a study modelling elimination in the Malian and Senegalese foci of [12, 13], which discussed the possibility of (protracted) recrudescence in the River Gambia focus based on EPIONCHO projections [41]. Other epidemiological studies in Kaduna State in Nigeria [42] and the Abu Hamed focus in Sudan [43] have reported interruption of transmission as a result of CDTI. Similarly, an international team of experts evaluated CDTI programmes which, between 2008 and 2014, provided ivermectin for at least 6 years; results from 12 countries showed that in areas with adequate annual ivermectin treatment coverage, satisfactory progress was made towards elimination and that 33 evaluation areas with a total population of 28 million people were close to, or had already reached, elimination [44]. In other (East African) foci, interruption of transmission has been achieved through the disappearance of the local *S. neavei* vector [45], or by a combination of long-term ivermectin distribution and vector elimination [46].

### Why onchocerciasis control remains difficult in certain areas

#### Issues affecting coverage and access to treatment

Achieving consistently high ivermectin treatment coverage remains a challenge and in several African countries, such as the Democratic Republic of the Congo (DRC), Central African Republic (CAR), Angola, Cameroon and South Sudan, onchocerciasis elimination may be out of reach in the near future [23]. Onchocerciasis control has been difficult in those African areas with initial prevalence greater than 60%, especially if ivermectin is only distributed once a year. This is mainly due to the fact that higher endemicity levels require higher coverage and longer treatment durations. For example, by 2015, after 15 years of CDTI, onchocerciasis was reported to remain mesoendemic in the Centre and Littoral Regions of Cameroon [47]. In the North and North-West of the country, the prevalence of onchocerciasis had dramatically decreased after 17 years of CDTI but elimination has certainly not yet been reached [48]. In the DRC not even the target coverage for onchocerciasis morbidity control (of 65%) had been reached by 2012 [49], let alone the target coverage for elimination (80%).

Barriers to access to treatment and poor treatment compliance contribute to insufficient treatment coverage. Although ivermectin is provided free of charge, several onchocerciasis endemic regions still do not have good access to treatment. For example, people may not receive their annual treatment because of inadequate supply as a result of underestimation of population size [50], or because the community directed distributors (CDDs) of ivermectin do not visit remote and inaccessible areas. In Cameroon, the number of CDDs available to cover several large villages and zones was deemed too small [48]. In Tanzania, lack of comprehensive understanding of the disease, fears of medication, distrust of the method determining dose, lack of health education materials, insufficient CDD-resident communication, and inflexible drug distribution mechanisms were identified as factors affecting community participation in the CDTI programme [51]. In South Sudan the ivermectin distribution campaign has been disturbed by the war. Moreover, health care providers working in remote endemic zones may fail to diagnose onchocerciasis due to insufficient training and poor resources [52] and as a result, endemic zones where ivermectin needs to be distributed may be missed.

Political restructuring and inadequate assessment of onchocerciasis endemicity can contribute to reduced coverage. For example, in the Ituri Province in the DRC, the lack of implementation of a CDTI programme in certain villages was caused by the reorganization of the health territory which resulted into the subdivision of health zones into several health areas. The villages of Bessi, Draju, Kanga, Ndroyi, and Wala, in the past belonged to the health zone of Angumu (where, based on rapid epidemiological mapping of onchocerciasis (REMO) assessment, CDTI was needed). Later they were integrated into the Logo health zone, a zone where CDTI was considered not to be necessary and, therefore the population in these villages did not receive ivermectin (M. Mandro, pers. comm.).

In the majority of CDTI projects in Africa, reported coverage has been satisfactory and, by and large, increasing over time [44]. However, ivermectin coverage is commonly calculated using the information provided by CDDs and such estimates can easily lead to over-estimation of coverage (particularly if population censuses are not regularly updated and the CDDs treat an increasing number of residents as populations grow but the denominators remain the same). Furthermore, coverage rates in a community may give a misleading picture of the success of control efforts; if there are individuals or large groups who systematically do not comply with treatment, they may provide a continued focus for transmission [26, 28, 31–33, 53] and make elimination of onchocerciasis an unattainable goal.

#### Issues affecting treatment adherence

In addition to inadequate access to treatment, onchocerciasis control efforts are further limited by poor compliance and uptake of ivermectin in some communities, among other factors, due to seasonal migration of workers at the time of ivermectin distribution, lack of incentives for CDDs, fear of side effects and distrust of CDDs [32]. Similar observations were made in Mahenge in Tanzania, where it was reported (based on a household-based survey) that during the annual 2016 CDTI round, the majority of community members were away for farming; besides, in this locality fear of side effects was one of the main reasons for not taking ivermectin (B. Mmbando, unpublished data). Women in particular were more often non-compliers because of fear of sterility [32], and since less than 25% of CDDs are female [25], increasing this proportion may inspire women’s confidence in taking the treatment. In Cameroon, but something also observed in the DRC, certain people do not take the ivermectin orally because they use ivermectin to kill hair lice [32]. In a village in the Bas Uele province of the DRC with a very high prevalence of epilepsy and high exposure to *Onchocerca*-infected blackflies, people had stopped taking ivermectin because of experiencing side effects and, according to information gathered during four focus-group discussions, having to pay for the treatment of these side effects (A. Rotsaert, unpublished data).

Treatment compliance has been associated with being male [50], living in an area for a longer time, and having social support [48]. In some settings, older age is associated with ivermectin uptake [50], whilst in others younger people are more likely to have taken ivermectin [49]. Positive beliefs about ivermectin that have been associated with treatment compliance include beliefs that ivermectin prevents onchocerciasis and blindness [48], induces intestinal worm expulsion, and increases vitality [47]. Perceived personal risk of onchocerciasis [54, 55] and positive perceptions of the programme have also been associated with good treatment adherence, and those that perceive CDDs as doing their work well, or know at least one CDD in their village, are more likely to take treatment [56].

Ivermectin treatment in loasis co-endemic areas presents one of the most important challenges [32]. Although considered a “safe” drug, administration of ivermectin to patients with both, onchocerciasis and loiasis, can result in severe adverse events (SAEs), including encephalopathy and death [57]. Early identification and referral of cases of encephalopathy to a hospital to provide medical and nursing care is of paramount importance. Not surprisingly, a fear of SAEs is a major reason for non-compliance in onchocerciasis-loiasis co-endemic areas.

#### Sub-optimal responses to ivermectin

Studies in Ghana and Cameroon suggest the occurrence of the so-called sub-optimal (or atypical) responses to ivermectin. In these studies ivermectin still killed the microfilariae but seemed to have become less effective in reducing the fertility of the adult female worms [58]. This resulted in a rapid reappearance of microfilariae and an increased risk of onchocerciasis transmission even when the frequency of treatment had been increased from annual to biannual [59]. These, essentially phenotypic, studies have been recently complemented by genome-wide analysis of ivermectin responses by *O. volvulus* [60]. This analysis suggests that the evolution of sub-optimal responses occurs via selective sweeps of pre-existing quantitative trait loci rather than via selection of relatively rare resistance-conferring mutation(s). The outcome is the accumulation of many alleles in a limited number of functional pathways that facilitate the recovery of adult female worm fecundity from the inhibitory effects of ivermectin. This is consistent with the observation that the microfilaricidal effect of ivermectin remains unaltered in sub-optimally responding populations, but that the difference between these and fully ivermectin-susceptible parasites resides in quantitative variation in the rate and extent to which microfilarial production is resumed after treatment [60].

#### Cross-border issues

Cross-border onchocerciasis transmission is another challenge for onchocerciasis control programmes that focus only on a narrowly-defined geographical area. Parasites can be reintroduced into an area where CDTI has good geographic and therapeutic coverage by immigrant humans (including refugees or seasonal migrants) or vectors from insufficiently controlled areas. Different vector species differ in their propensity to disperse and migrate. For example, *S. neavei* in Uganda is not known to disperse further than a few kilometres from its breeding sites, whereas *S. damnosum s.str*. can migrate (wind-assisted) up to 400 km in West Africa and carry parasites into controlled areas [30]. Human and vector migrants can carry parasites across national borders and between foci within a country. The WHO recommends the use of transmission zones as the units of assessment because they are expected to be epidemiologically independent from each other as migration between them is deemed to be negligible [21]. The mapping of transmission zones is, therefore, important for onchocerciasis elimination, but also problematic because patterns of migration (of vectors and humans) will be unique to each area and are very difficult to quantify and to map. One possibility may be through the use of parasite genetic markers to understand patterns of gene flow between populations, and recent advances in genomic analyses of *O. volvulus* may facilitate this [60].

### Suggestions to improve weak onchocerciasis elimination programmes (Fig. 1)

#### Areas of insufficient onchocerciasis control need to be identified

Such areas are generally located in hard-to-reach areas and populations, including in insecure areas, and there is little information about the status of onchocerciasis control in those settings. In South Sudan, for example, the CDTI programme seems to have been interrupted and there is no recent information on the onchocerciasis situation in this country. Challenges in accessing remote or conflict-affected areas, combined with poor resources, mean that there is also the need for revision and development of methodologies that will enable rapid, reliable and cost-effective assessment of the onchocerciasis control situation. However, this is not exclusively an issue for hard-to-reach and insecure areas. Five of the six APOC evaluation areas that were identified by Tekle et al. [44] as having unsatisfactory treatment coverage had no accessibility or security problems, highlighting the importance of monitoring and evaluation in all areas. A high prevalence of epilepsy, and certainly a high incidence of new onset epilepsy in children and youngsters between the ages of 3 and 20 years, in an onchocerciasis endemic area should be a reason to assess the performance of the CDTI programme. In non-onchocerciasis endemic regions in Africa most of the seizures in persons with epilepsy start below the age of 5 years because of obstetric and perinatal problems. In highly onchocerciasis-endemic regions, a large number of individuals may present with seizures after the age of 5, with a peak onset of epilepsy between the age of 8 and 12 years. The latter type of epilepsy should be considered as an early-warning sign of onchocerciasis-associated epilepsy (OAE) (Table 1). Interestingly, the study by Walker et al. [5] reported that for a given microfilarial load, the relative risk of mortality was significantly greater in children (aged < 20 years) than in those aged 20 years and more.

**Fig. 1:**
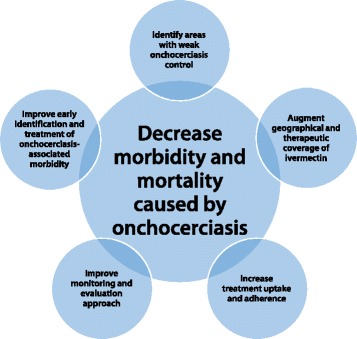
Improving weak onchocerciasis (oncho) elimination programmes. At the core of this effort is the recognition that some of these programmes may not reach elimination goals in the 2020/2025 time horizons but, if strengthened, they can still achieve substantial reductions in morbidity and mortality due to onchocerciasis. This requires (clockwise) the identification of under-performing programmes and investigation of the causes for this, with particular emphasis on improving the geographic (and therapeutic) coverage as well as treatment uptake and compliance. Monitoring and evaluation approaches should be improved with optimised use of current and novel tools; one such tool could be the recognition of early stage morbidity (e.g. prevalence of epilepsy in children) linked with serological markers of exposure

**Table 1:** Onchocerciasis-associated epilepsy (OAE), challenges and opportunities

New findings	Challenges	Opportunities
Burden of disease caused by onchocerciasis is more important than previously estimated	Accurate estimation of burden of disease due to onchocerciasis, including OAE, is a pressing need	Determination of OAE prevalence and incidence provides an argument to strengthen and accelerate onchocerciasis elimination programmes by identifying areas of weakness
	OAE awareness and advocacy are inadequate	Determination of OAE prevalence and incidence provides an argument to obtain more funding for operational research for onchocerciasis elimination efforts
High prevalence/ incidence of OAE suggest ongoing onchocerciasis transmission	Strengthen epilepsy surveillance in onchocerciasis endemic regions	CDDs could be engaged in assisting with epilepsy surveillance
OAE is preventable	Biannual CDTI should be promoted	Message will increase the motivation of populations to take ivermectin, potentially increasing compliance
	Misconceptions and stigma associated with epilepsy	Health promotion activities to reduce misconceptions and stigma among populations
OAE is treatable	In onchocerciasis-endemic regions, a decentralised system is needed to diagnose and treat epilepsy early and appropriately	CDDs could be trained to monitor antiepileptic treatment adherence
	Little collaboration between onchocerciasis elimination and mental health programmes	Onchocerciasis and public mental health programmes working together

*CDDs* community drug distributors, *CDTI* community directed treatment with ivermectin, *OAE* onchocerciasis-associated epilepsy

#### Geographical coverage of ivermectin needs to be improved and adapted to different contexts

Ivermectin distribution strategies deployed in conflict-affected areas may need to be different from the classical approaches to distribution in non-conflict settings. This could be done through collaboration with local NGOs or international humanitarian organizations such as International Red Cross, whose volunteers are often present in war zones. Training additional CDDs and providing suitable means of transportation in difficult terrain may help to ensure that more people living in remote areas have access to ivermectin.

#### Treatment uptake and adherence need to be improved through sustainable community participation

An effective social marketing campaign raising awareness about ivermectin, onchocerciasis and onchocerciasis-associated morbidities (including OAE) may motivate people to take up treatment and also improve adherence. Social research on people’s attitudes and perceptions regarding ivermectin and onchocerciasis can be used to advise on advocacy implementation strategies and to identify contextually relevant messages to be used for advocacy campaigns. Population groups with poor treatment uptake and the reasons for this need to be identified. Social science-based research can identify those strategies that, in a given context, will help the shift from insufficient CDTI coverage and adherence to well-performing CDTI programmes.

#### Timing of CDTI rounds should be improved

Ivermectin distribution campaigns need to be well-planned to take place at a time that ensures that the drug is effectively deployed during the main parasite transmission seasons (if transmission is highly seasonal), while taking into account when people are likely to be present in their communities and available for receiving the treatment (e.g. not during farming/harvesting periods). If increased treatment frequency is implemented, then it is important that treatment is administered 6-monthly to effectively curtail the transmission to blackfly vectors of microfilariae reappearing in the skin. This requires good coordination of the distribution of the drug from central points of arrival and storage to the districts and communities. Providing treatment twice a year but without the necessary interim period of 6 months between treatment rounds negates the benefits of biannual treatment [33].

#### Representation of women among community distributors should be increased and the motivation and training of the CDDs should be monitored and maintained

Studies report that female CDDs may strengthen and improve performance of the CDTI programme, as women are more committed, persuasive, more patient and their reports are more accurate [61]. Also, they may help to dispel misunderstandings about treatment and infertility among the women of the communities. However, a study in Uganda showed that it might be difficult for female CDDs to work effectively outside their own kinship zones and, also, that they may face more mistrust from the community than their male counterparts [62].

The motivation of CDDs and the empowerment of communities are essential for the success of CDTI. Therefore, CDTI needs to be tailored to adapt to local power structures and diverse cultural contexts. For instance, in Uganda traditional social systems are very strong in all rural communities; a kinship-enhanced CDTI strategy that adopts collective decision-making by community members was found to be more effective in achieving better treatment coverage and community participation than a classic CDTI approach, in which decisions are made primarily by community leaders without much involvement of community members [63, 64]. Improvement in treatment coverage observed in Uganda was largely attributable to involvement of kinship groups, avoidance of paying monetary incentives to the CDDs and the satisfaction with the programme of those who had been treated [63–65].

Maintaining commitment and motivation of CDDs is challenging, and reduced motivation may contribute to under-performance of CDDs. An appropriate form of compensation for CDDs largely depends on the context in which community leaders must agree to set their own terms of remuneration or locally appropriate incentives (e.g. currency, food or labour). In south-eastern Nigeria, lack of monetary incentives led to significant increases in CDD attrition [66], but in Plateau State the provision of monetary incentives to CDDs resulted in several problems, including complex logistics and making the position so desirable that community leaders often chose friends and relatives for the job [67]. By contrast, in Uganda, a shift from in-kind payments towards monetary-oriented strategies helped to achieve adequate drug distribution in Kabarole district [68]; in contrast, avoidance of paying monetary incentives to the ivermectin distributors contributed to improved treatment coverage in ten other districts [65].

Compensation and motivation of CDDs is very much a local issue based on the value judgements of the CDDs. When decisions regarding CDTI are made collectively, by community members rather than by community leaders and health workers on behalf of the community members, the CDDs’ demands for monetary incentives decline [69] but, on the other hand, CDDs who work among non-relatives are more likely to demand monetary incentives than those who treat relatives [63].

#### Treatment frequency should increase to biannual wherever possible, particularly in highly endemic areas

Regarding treatment frequency, biannual ivermectin distribution has been shown to improve treatment uptake [59], provide at least one treatment round in the year to those who may have missed the previous round, and shorten the timeframes to elimination by reducing the transmission of microfilariae to vectors in the inter-treatment periods [26–28], proving to be cost-effective [33]. Therefore, weak onchocerciasis control programmes, in particular, should be supported not only to increase their geographic and therapeutic coverage but also to implement biannual ivermectin distribution.

#### Approaches to the monitoring and evaluation of onchocerciasis control programmes should be improved

With ESPEN’s focus on onchocerciasis elimination, there is great need for improved approaches to the monitoring and evaluation of onchocerciasis control efforts. In 2016, the WHO published guidelines about how to make decisions concerning the stopping of CDTI and the evidence required for verification of interruption of transmission [34], but these guidelines do not provide enough information to advise countries as to how to monitor progress towards elimination. To this end, WHO has now created a working group to develop a programme managers’ guide.

The design of robust monitoring and evaluation activities and the preparation of clear guidance on these, including which data should be collected by programmes, are the subject of ongoing statistical and transmission dynamics modelling work. The various strands of this work include: i) refinement of sampling protocols (e.g. for parasitological, serological and entomological assessments with current tools); ii) incorporation as model outputs of potential additional diagnostic tools (e.g. novel markers of female worm reproductive activity); iii) use of statistically robust approaches for analysis and interpretation of results; iv) refinement of evaluation criteria and thresholds for safe cessation of mass treatment and verification of elimination; v) determination of optimal duration of post-CDTI and post-elimination surveillance periods; and vi) formulation of recommended strategies if achieving elimination proves difficult, or if infection is reignited or reintroduced [26, 28, 41, 70, 71].

##### Parasitology

Evaluation of progress can make use of skin snip surveys (still the gold standard for diagnosing active infection), and can be conducted in parallel with Ov16 seroprevalence surveys. However, if skin snips are used, particularly for epidemiological evaluations conducted after prolonged CDTI with good coverage, it is recommended to test snips using PCR-based methods to increase test sensitivity. Coverage surveys may not necessarily be the best indicators of onchocerciasis control programmatic effectiveness for the reasons discussed above and because of the insidious impact of systematic non-compliance. Albeit not of true diagnostic value for the determination of individual infection status, seroprevalence surveys using Ov16, especially if conducted at various time points after the start of CDTI, can provide important data to understand temporal (and spatial) trends in exposure patterns. In particular, the serological testing of children aged ≤10 years could be used as a tool for evaluating the performance of CDTI programmes (Table 2).

**Table 2:** Advantages and disadvantages of currently available tools for monitoring and evaluation of onchocerciasis control and elimination programmes

Monitoring tools	Advantages	Disadvantages
Skin snip surveys during the treatment implementation phase	Detection of skin microfilariae is the gold-standard diagnostic of active infection. PCR can be used on skin snips	Need ethical approval^*^; painful; require sterilisation of punches between individuals being sampled; decreasing acceptance by communities
Ivermectin coverage surveys	Relatively easy and affordable; can provide information about treatment uptake	May lead to overestimation of coverage and/or provide incomplete information about treatment adherence
Ov16 rapid diagnostic test (RDT) surveys in children aged up to 10 years	Relatively affordable, immediate answer on site	Need ethical approval^*^, sensitivity and specificity of RDTs not yet well established
Ov16 ELISA surveys in children aged up to 10 years	Sensitivity of up to 80% and specificity of up to 97% [72]	Need ethical approval^*^; more expensive than RDTs; samples need to be sent to a lab, often located abroad. Variability in diagnostic performance according to lab and presence of other filarial infections [92]
PCR pool screening of simuliid vectors	No ethical approval needed?^*^; many flies can be sampled; in principle, separate analysis of flies’ heads and bodies can provide information on infectivity to and from human populations	Lack of trained entomologists and labs, as samples often shipped to reference labs for PCR analysis; increasing number of flies needed as infection levels decrease; sampling protocols need to be refined

^*^Some ministries of health have given blanket ethical approval for all monitoring and evaluation activities (including skin snips, blood tests and catching flies by human vector collectors), as part of the control programme activities. Others seem to require approval for specific instances

##### Serology

Testing for Ov16 can be done with an enzyme-linked immunosorbent assay (ELISA) method or with the newly developed rapid Ov16 diagnostic test. The rapid diagnostic test (RDT) has a lower sensitivity and specificity than the ELISA test, but is cheaper, easier to perform and provides an immediate result onsite [72]. Ov16 point-of-care serosurveys in children aged up to 10 years have so far been used to decide whether onchocerciasis transmission has been interrupted [43, 45, 46], but they could also be used for programme evaluation. The WHO has proposed an Ov16 prevalence of 0.1% (upper confidence limit) in children below the age of 10 years as a suitable threshold for stopping ivermectin treatment [34], but in order to reach this threshold large sample sizes are required and the test specificity should be 100%. Recent modelling studies have sought to investigate the optimal age groups to be sampled under various scenarios of diagnostic performance using ONCHOSIM [70], while taking into account that a single threshold value may not be appropriate for all levels of initial endemicity (a similar problem arises with the pOTTIS not adequately reflecting the true underlying transmission breakpoints for different baseline endemicities) [26, 28]. Guidelines for using Ov16 serosurveys for programmatic performance evaluation would also need to be developed, and mathematical modelling can help in this endeavour.

As a case study, we performed a survey using the Ov16 RDT point-of-care test in the Mahenge area in Tanzania as part of a research project to study the relationship between the degree of onchocerciasis transmission and the incidence of epilepsy. A high prevalence of Ov16 seropositivity (41%; 95% *CI* = 34–48%) among children aged 7–10 years was observed in two villages that had a high prevalence of epilepsy (> 3%). In these villages, ivermectin had apparently been distributed for more than 19 years, and onchocerciasis had been considered to be well controlled according to reported treatment coverage data obtained during household surveys (in which about 76% of interviewed individuals stated having taken ivermectin during the previous year) (B. Mmbando, unpublished data). However, Tekle et al. [44] reported that the Mahenge focus had only 7 years of treatment with > 60% coverage by the time its evaluation by APOC was conducted, with a microfilarial prevalence of 10 – 45%, in agreement with our seroprevalence results.

How such Ov16 serosurveys should be performed in an ethical way as part of programme evaluation needs to be established. If, as seems likely, each country needs to obtain ethical approval for organising Ov16 serosurveys and if informed consent/assent needs to be obtained from each individual to be tested, the costs associated with the testing will be considerable.

##### Xenomonitoring

Molecular xenomonitoring using blackfly head pools (to detect infection by L3 larvae) has been proposed by the scientific international community for evaluation of impact of onchocerciasis elimination programmes as it provides information on parasite transmission from vectors to humans. As elimination programmes progress, testing for blackfly bodies (abdomens plus thoraces) could provide additional and useful indicators of transmission from humans to vectors (i.e. uptake of live skin microfilariae that would otherwise be difficult to detect by skin snips or skin-snip PCR [73]). It is paramount that molecular xenomonitoring be conducted as part of epidemiological evaluations as it complements information provided by skin snips or other techniques [74]. However, the technical expertise necessary to perform such entomological investigations in a satisfactory manner (designing well-suited sampling protocols; determining sample sizes, where to sample; when to sample; identifying biting simuliids to species, etc.) is currently lacking in many endemic countries.

Many entomologists with expertise in blackfly vectors have retired or moved to work in other, better funded fields, such as malaria. Therefore, there is an urgent need to train young African entomologists and motivate them to work on Simuliidae. Catching flies also poses the ethical problem of how best to do this. The recommended way is still by human landing capture, which also provides information on biting rates. In Ghana, we have tried a number of strategies, including host-dependent and host-independent catching methods [73, 75], and others have tried to develop and optimise (e.g. Esperanza window) traps that would obviate the need for human attractants [76, 77]. The advantage of human landing catches is their comparative value with the standardised methods used by the OCP [78]. Also they provide the possibility of estimating biting rates and infective biting rates/transmission potentials [73, 75] rather than just proportions of flies infected/infective (which devoid of the context of vector density are non-informative of transmission intensity). As long as the vector collectors are recruited locally and are taking regular ivermectin treatment, the procedure is considered not to be harmful. Ideally, however, as large fly population samples are needed, and the sample size required may increase with decreasing infection levels in the human population, more efficient, non-hazardous, and large-scale sampling methods will be necessary. Recently, it has been reported that Esperanza traps may be effectively operated by community residents and represent a viable alternative to human landing collections for entomological surveillance of *O. volvulus* transmission [79].

##### Ivermectin efficacy

If during regular programme evaluation issues with ivermectin uptake are identified, these should be picked up by the national elimination committees, which need to recommend corrective actions. These may include conducting a coverage verification study. If coverage is found to be satisfactory, human (and vector) migration studies may need to be undertaken. Parasite genetic studies may also need to be considered if Ov16 serosurveys suggest high levels of ongoing transmission despite long CDTI duration with good coverage. Studies need to investigate potential contributory factors to decreased ivermectin sensitivity and the impact that any potential ivermectin resistance may have on achieving onchocerciasis elimination [80]. Most ivermectin resistance studies had focused on candidate genes (e.g. beta-tubulin) identified in ivermectin-resistant nematodes of farmed ruminants (e.g. [81]). Consequently, modelling work had explored the spread of (recessive) resistance in one locus-two allele systems (e.g. [82]). However, the recent work using genome-wide approaches described earlier has revealed that the phenotype of sub-optimal response to ivermectin is likely determined by quantitative trait loci with many genes contributing in a polygenic manner [60]. Ongoing modelling studies are focussing on the impact of the latter upon onchocerciasis elimination (L.E. Coffeng, pers. comm.).

#### Treatment, care, and support for persons with onchocerciasis-associated morbidities should be enhanced

Because of the long-term onchocerciasis control programmes that have been in place (OCP, APOC, OEPA), the number of blind and visually impaired people due to onchocerciasis has decreased substantially as the incidence of infection has decreased (although prevalent cases of blindness still remain). The burden and psychosocial consequences of onchocercal skin disease have been well recognised [83, 84]. There is, however, still an important morbidity associated with onchocerciasis that has thus far been largely neglected by most health care systems, namely onchocerciasis-associated epilepsy (OAE). Onchocerciasis control programmes and burden of disease studies have not considered epilepsy among the sequelae of onchocerciasis (whose causal relationship is difficult to ascertain and thus most studies have been largely ecological [6–8]). This has resulted in the programmes not addressing this major public health problem and/or lacking the means of evaluating its burden and temporal/spatial evolution. Neurologists are not generally present in onchocerciasis-endemic regions and mental health initiatives only tend to consider (neuro) cysticercosis as the main parasitic disease causing epilepsy. It will become increasingly important that onchocerciasis control and mental health programmes work together. Thus both programmes should exchange surveillance data. Moreover, CDDs could play an important role in epilepsy surveillance systems, should these be established, in order to detect persons with new onset epilepsy early and refer them for treatment promptly. Even after implementing and strengthening effective onchocerciasis control programmes, those already affected will continue to suffer even after transmission has been interrupted.

OAE will potentially make the burden of onchocerciasis disease in Africa considerably greater than previously thought. Recent studies (in line with previous reports [85]) suggest that in onchocerciasis endemic regions, infection by *O. volvulus* may trigger epilepsy, with ivermectin effecting some protection against seizures [86–88]. This protection, however, may be only partial where ivermectin is given on an annual basis (because adult worms resume production of microfilariae after a few months). Further research needs to be conducted to ascertain the impact of ivermectin treatment on the incidence of epilepsy in prospective studies, but most likely biannual treatment will be necessary to suppress microfilarial load and potentially to have a maximal effect on the incidence of OAE. By eliminating onchocerciasis, it is anticipated that the incidence of OAE will decrease and with it, its burden of long-term disability. While technically, elimination strategies may not include OAE, it is important to document it, as it has an impact on compliance at the local level and is a major but underestimated factor of relevance for advocating the elimination of onchocerciasis.

#### Funding should be increased to support operational research and to help countries with weak onchocerciasis control to move towards elimination

In addition to ivermectin, the Alternative Treatment Strategies (ATS) document of APOC (2015) [21] also outlines the possibility of deploying other strategies (including focal vector control [71]) and therapies as they become available for safe use in humans (e.g. moxidectin; macrofilaricides in the pipeline) or when they are already available for other indications (e.g. doxycycline), as well as a number of test-and-treat options for areas co-endemic with loiasis, sub-optimal responses to ivermectin, or mop-up settings. In particular, co-endemicity with loiasis has represented a major impediment to the expansion and intensification of mass ivermectin treatment coverage and compliance for the reasons mentioned above. Inroads into such challenges have been made possible by recent technical advances in rapid loiasis diagnostics [89] for the identification of heavily microfilaraemic individuals at high risk of SAEs who would not be offered ivermectin. These, however, only represent a small fraction of the population (1–2%) [90]. This strategy has proven safe and effective for the implementation of district-wide, community-based distribution of ivermectin in loiasis–onchocerciasis co-endemic areas in Cameroon [91]. Large-scale implementation trials of these and other ATS require additional funding to evaluate not only their feasibility as a proof-of-concept but crucially their epidemiological impact and cost-effectiveness.

Countries and areas within countries that currently have under-performing onchocerciasis control programmes already suffer from under-staffed and under-resourced healthcare systems, and allocating scarce resources to improving onchocerciasis control may not be at the top of their public health agenda. It is imperative that OAE is well researched, its association with *O. volvulus* infection rigorously established and recognised, and that future burden of disease studies include its association with morbidity and mortality. We hope that recognising OAE as one of the most important onchocerciasis-associated morbidities and that assessing its burden on society will motivate public health decision makers to improve the performance and monitoring of CDTI programmes and also motivate funders to increase their support for actions towards the elimination of onchocerciasis.

## Conclusions

The long-term aim of programmes against onchocerciasis should remain the interruption of its transmission. It is likely, however, that in several African countries this goal will not be reached in the 2020–2025 timeframes proposed by WHO and APOC. In view of the challenges associated with onchocerciasis control, ESPEN’s mandate should be expanded to include the strengthening of weak onchocerciasis control programmes. However, one of the major hurdles for ESPEN will be to reconcile the need for increased activities around the other four preventive chemotherapy NTDs under its remit with the specific requirements of a Pan-African scale elimination programme. If onchocerciasis is to be eliminated in Africa, it will be crucial for ESPEN to engage promptly and collaboratively with the international scientific community and major international funders as well as with other stakeholders to harness the all-essential technical and financial support that will be necessary to address this huge endeavour, identify areas where CDTI programmes are performing less well than they should, determine the reasons for this, and find novel ways of monitoring, evaluating and supporting the programmes, as well as identifying which, where, and when ATS should be deployed, as they become part of our armoury in the fight against River Blindness.

## Additional file

Additional file 1 Multilingual abstracts in the five official working languages of the United Nations. (PDF 343 kb)

## Abbreviations

APOC: African Programme for Onchocerciasis Control
ATS: Alternative treatment strategies
CAR: Central African Republic
CDD: Community drug distributor
CDTI: Community-directed treatment with ivermectin
*CI*: Confidence interval
DALY: Disability-adjusted life year
DRC: Democratic Republic of the Congo
ELISA: Enzyme-linked immunosorbent assay
ESPEN: Expanded Special Project for the Elimination of Neglected Tropical Diseases
MSD: Merck Sharpe and Dohme
NGO: Non-Governmental Organization
NTD: Neglected tropical disease
OAE: Onchocerciasis-associated epilepsy
OCP: Onchocerciasis Control Programme in West Africa
OEPA: Onchocerciasis Elimination Program for the Americas
PCR: Polymerase chain reaction
pOTTIS: Provisional operational thresholds for treatment interruption and initiation of surveillance
RDT: Rapid diagnostic test
REMO: Rapid epidemiological mapping of onchocerciasis
*s.l.*: sensu lato
*s.str*.: sensu stricto
SAE: Severe adverse event
UK: United Kingdom
USA: United States of America
WHO: World Health Organization
