# Supplementary material for: From river blindness control to elimination: bridge over troubled water
Source: Infect Dis Poverty. 2018 Mar 28;7:21. doi: 10.1186/s40249-018-0406-7 (PMC5872540; doi:10.1186/s40249-018-0406-7)
Supplement: Supplementary file 1 — Multilingual abstracts in the five official working languages of the United Nations. (PDF 343 kb) [file 40249_2018_406_MOESM1_ESM.pdf]

Translation of the abstract into the five official working languages of the United Nations

داء كلابية الذنب من السيطره عليه للقضاء عليه: محاولة لتخطي العقبات

روبرت كولبوندرس ، ماريا-جلوريا باسانيز ، كاتيا سيلينج ، روري ج بوست ، أنك روتسايرت ، برونو ممباندو ، باتريك سويكيربويك [1]، أدريان هوبكنز

الملخص

خلفية: يقدر عدد المصابين بفيروس داء كلابية الذنب حاليا بنحو 25 مليون شخص (وهو عدوى طفيلية تسببها النيماتودا الفيلارية التي تنتقل عن طريق ناقلات العصابات)، و 99٪ منهم في أفريقيا جنوب الصحراء الكبرى. وقد أوقف البرنامج الأفريقي لمكافحة داء كلابية الذنب في ديسمبر 2015، وأنشأت منظمة الصحة العالمية هيكلا جديدا، وهو المشروع الخاص الموسع للقضاء على الأمراض المدارية المهملة من أجل تنسيق الدعم التقني للأنشطة التي تركز على خمسة أمراض مدارية مهملة في أفريقيا، بما في ذلك داء كلابية الذنب.

الأهداف: في هذه الورقة، نرى أنه على الرغم من ترسيم استراتيجية القضاء المحددة بشكل معقول، فإن تنفيذها سيتعرض لصعوبات خاصة عند الممارسة العملية. ونحن نهدف إلى إبراز هذه الأمور في محاولة لضمان فهمها على نحو جيد، ووضع خطط فعالة لحلها من جانب الدول المعنية وشركائها الدوليين.

الاستنتاجات: ثمة مصدر قلق محدد هو عبء المرض الذي يسببه الصرع المرتبط بمرض داء كلابية الذنب في المناطق التي توجد فيها فرط توطن للمرض في الدول التي تواجه صعوبات في تعزيز برامج مكافحة داء كلابية الذنب. وينبغي تحديد هذه الصعوبات ودعم البرامج أثناء الانتقال من مكافحة الأمراض إلى وقف انتقال المرض والقضاء عليه.

Translated from English version into Arabic by Mahmoud Sami, through

TRANSLATORS
WITHOUT BORDERS

河盲症控制与消除：解决疫水问题

Robert Colebunders, Maria-Gloria Basáñez, Katja Siling, Rory J Post, Anke Rotsaert, Bruno Mmbando, Patrick Suykerbuyk, Adrian Hopkins

摘要

引言：目前，约有 2,500 万人罹患盘尾丝虫病（由感染丝状线虫盘尾丝虫引发，经蚋媒传播），99%分布在撒哈拉以南的非洲。2015 年 12 月，非洲盘尾丝虫病控制项目结束，世界卫生组织组建了一个新项目——消除被忽视的热带病扩展特别项目，以协调对非洲五种被忽视的热带病消除行动的技术支持，其中包括盘尾丝虫病。

主要内容：本文认为，尽管制定了合理明确的消除策略，但该项目在实际实施中将会面临一些困难。本文旨在强调这些问题，确保他们能够得到充分认知，以敦促有关国家与其国际合作伙伴制定有效的计划解决这些问题。

结论：特别值得关注的是，高流行区盘尾丝虫病所致癫痫病引起的疾病负担加重了项目实施的难度。在从控制发病率到中止传播至消除过程中，应确定这些困难并开展相应的支持项目。

Translated from English version into Chinese by Jin Chen, through

CHINESE CENTER FOR DISEASE CONTROL AND PREVENTION
NATIONAL INSTITUTE OF PARASITIC DISEASES

Du contrôle à l'élimination de l’onchocercose (cécité des rivières): Comme un pont sur l'eau trouble

Robert Colebunders[1,*], Maria-Gloria Basáñez[2*,**], Katja Siling[3,4], Rory J Post[4,5], Anke Rotsaert[1], Bruno Mmbando[6], Patrick Suykerbuyk[1], Adrian Hopkins[7]

Résumé

Contexte : On estime que 25 millions de personnes sont actuellement infectées par l’onchocercose (une parasitose causée par le nématode filarien *Onchocerca volvulus* et transmise par des diptères du genre *Simulium*, mouches noires), dont 99 % en Afrique subsaharienne. Le Programme Africain de Lutte Contre l’Onchocercose s’est achevé en décembre 2015, et l’Organisation Mondiale de la Santé a mis en place une nouvelle structure, le Projet Spécial Élargi pour l’Élimination des Maladies Tropicales Négligées (ESPEN), chargé de la coordination du soutien technique aux activités visant en particulier cinq maladies tropicales négligées en Afrique, et notamment l’élimination de l’onchocercose.

Buts : Notre article montre que, bien qu’une stratégie d’élimination raisonnablement bien définie ait été ébauchée, sa mise en application pratique présentera des difficultés particulières. Nous souhaitons mettre en lumière ces dernières afin de nous assurer qu’elles soient bien comprises et que des plans efficaces puissent être mis en place afin que les pays concernés et leurs partenaires internationaux puissent les résoudre efficacement et durablement.

Conclusions : Une problématique particulière identifiée concerne le fardeau de l’épilepsie associée à l’onchocercose dans les zones d'hyperendémie situées dans des pays qui ont de la peine à renforcer leurs programmes de lutte contre l’onchocercose. Ces difficultés doivent être identifiées et ces programmes locaux soutenus pendant la transition du contrôle de la morbidité à l’interruption de la transmission et à l’élimination.

Translated from English version into French by Suzanne Assenat, through

TRANSLATORS WITHOUT BORDERS

От борьбы с речной слепотой к её ликвидации: преодоление препятствий

Роберт Коулбандерс[1,*], Мария-Глория Базаньес[2*,**], Катя Сайлинг[3,4], Рори Дж. Пост[4,5], Энке Ротсаерт[1], Бруно Ммбандо[6], Патрик Сайкербайк[1], Эдриан Хопкинс[7]

Аннотация

Краткое описание. По оценкам в настоящее время 25 миллионов человек инфицированы онхоцеркозом (паразитарной инфекцией, которая вызывается филярийными нематодами *Onchocerca volvulus* и переносится мошками *Simulium*), причём 99% инфицированных проживают в странах Африки к югу от Сахары. Программа по борьбе с онхоцеркозом в Африке была закрыта в декабре 2015 года, и Всемирная организация здравоохранения учредила новую структуру «Расширенный специальный проект по ликвидации недооценённых тропических заболеваний», предназначенную для координации технической поддержки деятельности, направленной на борьбу с пятью недооценёнными тропическими заболеваниями в Африке, включая ликвидацию онхоцеркоза.
Цели. В настоящей статье мы утверждаем, что, несмотря на формирование обоснованной и чёткой стратегии по ликвидации заболеваний, её практическая реализация связана с определёнными трудностями. Мы стремимся обозначить эти трудности, чтобы обеспечить их полное понимание и выработать эффективный план по их устранению вовлеченными в эту деятельность странами и их международными партнёрами.
Выводы. Особую озабоченность вызывает бремя болезней, связанное с эпилепсией, вызванной онхоцеркозом, в гиперэндемических зонах, которые находятся в странах, сталкивающихся с трудностями при развитии своих программ по борьбе с онхоцеркозом. Необходимо выявить данные трудности и оказать поддержку программам на этапе перехода от снижения показателей заболеваемости к борьбе с инфицированием и ликвидации заболеваний.

Translated from English version into Russian by Galina Dmitrieva, proofread by Natalia Potashnik, through

TRANSLATORS
WITHOUT BORDERS

Desde el control de la oncocercosis ("ceguera de los ríos") hasta su eliminación: Puente sobre aguas turbulentas.

Robert Colebunders, Maria-Gloria Basáñez, Katja Siling, Rory J Post, Anke Rotsaert, Bruno Mmbando, Patrick Suykerbuyk, Adrian Hopkins

Resumen

Antecedentes: Alrededor de 25 millones de personas padecen de oncocercosis (una infección parasitaria causada por el nematodo filarioideo *Onchocerca volvulus*, y transmitida por simúlidos (Diptera: Simuliidae), también conocidos como moscas negras), 99% de las cuales residen en África Subsahariana. El Programa Africano para el Control de la Oncocercosis (APOC) clausuró en diciembre de 2015 y la Organización Mundial de la Salud lanzó el Proyecto Especial Ampliado para la Eliminación de Enfermedades Tropicales Desatendidas (ESPEN) a fines de coordinar la asistencia técnica para actividades centradas en cinco enfermedades tropicales desatendidas en África, incluyendo la eliminación de la oncocercosis.

Objetivos: En este artículo sostenemos que, a pesar del trazado de una estrategia razonablemente bien definida para la eliminación de la oncocercosis, la implementación de dicha estrategia presenta, en la práctica, dificultades muy concretas. Nuestro objetivo es el de resaltar estas dificultades en un intento de asegurar que sean bien entendidas y que puedan diseñarse planes efectivos para que tanto los países afectados como sus aliados internacionales puedan resolverlas.
Conclusiones: Un aspecto importante es la carga de la enfermedad causada por la epilepsia asociada con la oncocercosis en las zonas hiperendémicas que están situadas en aquellos países en los que el fortalecimiento de los programas de control de la oncocercosis plantea dificultades. Estas dificultades deben ser identificadas y debe lograrse apoyo para los programas durante la transición desde el control de la morbilidad hasta la interrupción de la transmisión y la completa eliminación de la enfermedad.

Translated from English version into Spanish by irene1204, proofread by Yaotl Altan, through

TRANSLATORS
WITHOUT BORDERS
